# *In vitro* Study of a Novel Stent Coating Using Modified CD39 Messenger RNA to Potentially Reduce Stent Angioplasty-Associated Complications

**DOI:** 10.1371/journal.pone.0138375

**Published:** 2015-09-18

**Authors:** Meike-Kristin Abraham, Andrea Nolte, Rebekka Reus, Andreas Behring, Diane Zengerle, Meltem Avci-Adali, Jan David Hohmann, Karlheinz Peter, Christian Schlensak, Hans Peter Wendel, Stefanie Krajewski

**Affiliations:** 1 Department of Thoracic, Cardiac and Vascular Surgery, Clinical Research Laboratory, University Hospital Tuebingen, Teubingen, Germany; 2 Atherothrombosis and Vascular Biology, Baker IDI Heart & Diabetes Institute, Melbourne, Victoria, Australia; University Hospital Medical Centre, GERMANY

## Abstract

**Background:**

Stent angioplasty provides a minimally invasive treatment for atherosclerotic vessels. However, no treatment option for atherosclerosis-associated endothelial dysfunction, which is accompanied by a loss of CD39, is available, and hence, adverse effects like thromboembolism and restenosis may occur. Messenger RNA (mRNA)-based therapy represents a novel strategy, whereby *de novo* synthesis of a desired protein is achieved after delivery of a modified mRNA to the target cells.

**Methods and Findings:**

Our study aimed to develop an innovative bioactive stent coating that induces overexpression of CD39 in the atherosclerotic vessel. Therefore, a modified CD39-encoding mRNA was produced by *in vitro* transcription. Different endothelial cells (ECs) were transfected with the mRNA, and CD39 expression and functionality were analyzed using various assays. Furthermore, CD39 mRNA was immobilized using poly(lactic-*co*-glycolic-acid) (PLGA), and the transfection efficiency in ECs was analyzed. Our data show that ECs successfully translate *in vitro*-generated CD39 mRNA after transfection. The overexpressed CD39 protein is highly functional in hydrolyzing ADP and in preventing platelet activation. Furthermore, PLGA-immobilized CD39 mRNA can be delivered to ECs without losing its functionality.

**Summary:**

In summary, we present a novel and promising concept for a stent coating for the treatment of atherosclerotic blood vessels, whereby patients could be protected against angioplasty-associated complications.

## Introduction

Atherosclerosis is the most common inflammatory disease of arterial vessels, which can lead to life-threatening myocardial infarction or ischemic stroke [1]. Its characteristics include the accumulation of lipids, cells and connective tissue inside the inner layer of the arterial wall. The following pathology is characterized by disturbed laminar flow, elasticity loss and chronic inflammatory processes such as the activation and adhesion of platelets [1,2]. The causes of this vascular disease are multifaceted and include obesity, smoking, hypertension, age, diabetes and hereditary conditions [3].

Coronary angioplasty, the common treatment for atherosclerotic vessels, is a minimally invasive procedure in which a bare-metal stent (BMS), a drug-eluting stent (DES) or a biodegradable stent is inserted into the narrowed vessel [4]. However, stent angioplasty interferes with the physiological state of the blood vessel. Due to the implantation of foreign material, the anti-thrombotic properties of the endothelium can be further destroyed, which leads to inflammatory processes like the activation of platelets accompanied by the release of nucleotides such as adenosine diphosphate (ADP) and adenosine triphosphate (ATP) [5]. A generated positive feedback loop (autocrine activation) induces the activation of additional platelets. Hence, anti-platelet therapy is required following stent angioplasty, which prevents thrombosis and thus vessel occlusion, but also increases the risk of hemorrhagic complications [6].

Extracellular nucleotides are also associated with the development of intimal thickening by stimulating the proliferation of vascular smooth muscle cells (VSMC) after stent angioplasty [5,7].

In comparison with the still frequently used BMSs, the application of DESs is superior in reducing in-stent restenosis after angioplasty. However, DESs are also not able to protect all patients from late in-stent restenosis. They also have certain side effects such as delayed healing, endothelial dysfunction and stent mal apposition [4,8].

The ecto-nucleoside triphosphate diphosphohydrolase 1-(ENTPD-1), also called CD39, is described as a promising anti-thrombotic therapeutic agent [9–12]. CD39, which is membrane-bound and constitutively expressed on intact endothelial cells (ECs), hydrolyzes ATP and ADP thereby preventing platelet activation and recruitment as well as maintaining blood fluidity. Additionally, in synergy with CD39, the ecto 5´nucleotidase CD73 cleaves adenosine monophosphate (AMP) to adenosine, an immunosuppressant. Its antithrombotic effects prevent the proliferation of smooth muscle cells and stimulate proliferation of endothelial cells [13].

Previous studies also showed that the administration of a soluble form of CD39 (solCD39) reduces neointimal hyperplasia after an induced vessel injury in mice [12].

The aim of our study is to develop and investigate a novel stent coating, which can reduce stent angioplasty-associated complications. Previous studies have shown an enormous potential for the exogenous delivery of synthetic messenger RNA (mRNA) as a therapeutic agent in the fields of regenerative medicine and treatment of diseases. This therapy is based on the delivery of a specific modified mRNA to the target cells, which then in turn express the desired proteins after mRNA translation [9,14]. Moreover, the mRNA-based therapeutic strategy has significant advantages: (1) the introduced mRNA is translated by the cell's own translational machinery under physiological conditions; (2) by introducing a specific mRNA, the physiological state of the cell is not altered, since the effect is transient and not mutagenic; (3) the protein synthesis can be controlled directly without intervening in the human genome; (4) the mRNA does not need to enter the nucleus for translation. Overall, this therapeutic strategy could be safely used in patients and is more cost effective and easier to manipulate than DNA-based gene therapy [15,16].

We hypothesize that by inducing the overexpression of CD39 at the site of the implanted stent, a decrease in platelet activation and also a reduction in restenosis and inflammatory responses following stent implantation may be achieved. Therefore, a modified synthetic mRNA encoding for CD39 was generated and the transfection efficiency and functionality of the expressed protein was investigated in human embryonic kidney cells 293 (HEK293) cells, immortalized porcine endothelial cells (PAE) and primary human ECs. The inhibition of platelet activation by the expressed CD39 protein was further analyzed. Moreover, a preliminary stent coating involving PLGA as a carrier for the CD39 mRNA was established and the transfection of cells mediated by the coating was analyzed.

## Materials and Methods

### mRNA generation

CD39 and enhanced green fluorescence protein (eGFP) mRNAs were generated as previously described [14]. Briefly, the pcDNA 3.3 plasmid (Eurofins Genomics GmbH, Ebersberg, Germany) containing CD39 or eGFP DNA templates was amplified using the HotStar HiFidelity Polymerase Kit (Quiagen, Hilden, Germany) according to the manufacturers instructions. To amplifiy the gene, two primers were used: TTGGACCCTCGTACAGAAGCTAATACG as forward primer and T_120_CTTCCTACTCAGGCTTTATTCAAAGACCA as reverse primer (Ella Biotech, Martinsried, Germany). The PCR product (PCR cycler: Eppendorf, Wesseling, Germany) was purified with the Qiaquick PCR Purification Kit (Qiagen) and eluted in 2 x 10 μl ddH_2_O (Qiagen).

*In vitro* transcribed mRNA was generated with the MEGAscript® T7 Kit (Ambion, Glasgow, Scotland) according to the manufacturers instructions. To modify the mRNA a 3´-0-Me-m^7^G(5')ppp(5')G RNA Cap Structure Analog (New England Biolabs, Frankfurt, Germany) was added to the reaction as well as pseudouridine-5'-triphosphate and 5-methylcytidine-5'-triphosphate (TriLink Biotech, San Diego, CA, USA), which were substituted for UTP and CTP, respectively. For RNase inhibition 1 μl of RNase inhibitor (Themo Scientific, Waltham) was added per reaction.

The *in vitro* transcriped mRNA was purified with the RNeasy Kit (Qiagen) and eluted in 2 x 15 μl ddH_2_O (Qiagen). Afterwards, the mRNA was dephosphorilized using the Antarctic Phosphatase Kit (New England Biolabs) and once again purified with the RNeasy Kit (Qiagen) in 2 x 40 μl ddH_2_O. The mRNA fragments were checked for quality and purity on a 1% agarose-TBE gel and the concentration of the mRNA was adjusted to 100 ng/μl with ddH_2_O.

### Transfection of HEK293 cells, immortalized porcine endothelial cells and primary human endothelial cells

Isolation of primary human ECs from residual saphenous vein biopsies was performed as previously described and approved by the ethics committee of the University of Tuebingen, Tuebingen, Germany [17]. One day prior to the transfection, HEK293 cells and human ECs were seeded with a density of 150.000 cells per well onto a 12-well plate (Corning Costar, NY, USA). For transfection of the PAE cells (SibTech, Brookefield, USA) the double amount of cells were seeded and hence twice the amount of the following transfection components were used. For mRNA complexiation, the mRNA was incubated with 2 μl Lipofectamine 2000 (Invitrogen, Glasgow, Scotland) and 500 μl Opti-MEM I (Invitrogen, Glasgow, Scotland) for 20 min at room temperature. After the cells were washed with 1 ml PBS (w/o Ca^+^/Mg^+^; PAA, Pasching, Austria), the transfection solution was added to the cells and incubated at 37°C and 5% CO_2_ (Heraeus 6000 Thermo Fischer Scientific, Langenselbold, Germany). After 4 h, the transfection solution was removed, the cells were washed with PBS (w/o Ca^+^/Mg^+^) and 1 ml of cell culture medium was added to the cells.

### Flow cytometry and fluorescence microscopy

Flow cytometric analyses of the transfected ECs, HEK293 and PAE cells was performed to detect CD39 surface expression. Thus, 24 h, 5 days or 7 days after transfection, cells were washed with PBS (w/o Ca^+^/Mg^+^) and split into two 50 μl aliquots. One aliquot of each sample was stained with 5 μl anti-CD39-fluorescein isothiocyanate (FITC) antibody (Abcam; Clone A1) or 5 μl anti-CD39-phycoerythrin (PE) antibody (BD Bioscience) for 20 min at room temperature in the dark, whereas the second sample was incubated with 5 μl PBS (w/o Ca^+^/Mg^+^) as control. After antibody staining, cells were washed and fixed using 250 μl 1x CellFix (BD Biosciences). Flow cytometry was performed within 6 h using a FACScan cytometer (BD Biosciences, USA) and a total of 10.000 events were acquired in each sample.

CD39 expression of HEK293 cells was also analyzed under a fluorescence microscope. Therefore, the cells were fixed with 500 μl 1x CellFix (BD Biosciences) and incubated for 15 min at room temperature. Afterwards, cells were washed twice and stained with 10 μl anti-CD39-FITC antibody (Abcam, Clone A1) diluted in 500 μl PBS for 20 min at room temperature. Again, the cells were washed twice with PBS and fluorescence microscopic analyses were performed using an inverse microscope (Axiovert 135, Zeiss, Germany).

### Detection of free phosphate and platelet activation

CD39 mRNA transfected HEK293 cells and ECs were incubated with 20 μM ADP (möLab, Langenfeld, Germany) or PBS as control for 10 min at 37°C on a shaking platform (Polymax 1040, Heidolph, Schwabach, Germany). Afterwards, free phosphate was measured in the supernatant using the Malachite Green Phosphate Assay Kit (BioAssay Systems) according to the manufacturer´s instructions.

Furthermore, after incubation with HEK293 cells or ECs 200 μl of the ADP superantant was incubated with 4 μl of platelet-rich plasma (PRP) for 10 min at 37°C. Blood sampling procedures were approved by the ethics committee of the University of Tuebingen, Germany (approval number 270/2010 BO1). The blood was collected from six healthy volunteers, who gave signed informed consent and was anticoagulated with 1.5 IU/mL heparin-sodium (Rathiopharm GmbH, Ulm, Germany). PRP was prepared as previously described [18].

After the incubation, platelets were either incubated with an anti-CD62P-PE antibody (BD Biosciences), with an anti-CD62P-PE antibody and additional ADP (20 μM) or with PBS at room temperature for 15 min. Platelets were then fixed with 350 μl of 1 x Cellfix (BD Biosciences) and measured using a FACScan cytometer.

### MTT-Assay

The viability of transfected HEK293 cells was verified by the MTT (3-(4,5-dimethylthiazol-2-yl)-2,5-diphenyltetrazolium bromide, AppliChem, Darmstadt, Germany) assay. Therefore, 150.000 HEK293 cells per well were seeded in a 12-well plate and transfected with different concentrations of CD39 mRNA. After 24 h and 48 h the cells were washed with RPMI (without phenol red, life technologies), trypsinized and 50.000 cells of each sample were incubated with 250 μg MTT dissolved in RPMI (without phenol red) for 4 h at 37°C. Afterwards, the MTT solution was removed and the cells were incubated with 300 μl DMSO (dimethyl sulfoxide, Serva, Heidelberg, Germany) for 10 min at 37°C. The absorbance was measured at 540 nm with the Mithras LB 940 Microplate Reader (Berthold technologies, Bad Wildbad, Germany).

### Cytokine assay

The release of cytokines and chemokines was measured using the Proteome Profiler^TM^ Array (Human Cytokine Array Panel A, R&D Systems, USA) Therefore, 300.000 HEK293 cells were seeded into a 6-well plate and transfected with different amounts of CD39 mRNA 24 h later. After another 24 h, a variety of cytokines were detected in the supernatants according to the manufacturer’s instructions. Data analysis was performed by measuring the pixel density in each spot of the array and calculating the data with the image processing program ImageJ.

### Coating of thermanox plastic slides with CD39 mRNA and PLGA

The stent coating was simulated using thermanox plastic slides (Nunc, Thermo scientific, USA). First, 100.000 HEK293 cells per well were seeded on a 12-well plate. After 24 h, 2 μl Lipofectamin as well as 10 μg mRNA were mixed with 50 μl Opti-MEM and incubated at room temperature for 20 min. Meanwhile, 10 μl from a PLGA (Evoniks, Darmstadt) stock solution (20 mg/ml)) were diluted in 990 μl ethyl acetate (final concentration 200 μg/ml). Then 200 μl of the PLGA solution were mixed with the transfection complexes. The thermanox slides were coated with the solution in a step-by-step approach at room temperature. eGFP mRNA and H_2_O dest. were used as controls. The HEK293 cells were supplied with new medium before the dried slides were plated face down onto the cells. The cells were incubated with the slides at 37°C and 5% CO_2_ for 24 h, 48 h and 72 h and then analyzed using a FACScan cytometer.

### Statistics

Data are depicted as means with standard error of the mean (SEM). Data were analyzed using one-way or repeated measures (RM) ANOVA with Bonferroni’s multiple comparison test to analyze differences between groups.

All analyses were performed using the statistical software package GraphPad Prism (version 5, GraphPad Software, La Jolla, USA). Statistical significance was defined as p < 0.05.

## Results

To prevent platelet activation and restenosis as well as to maintain the vessel wall biology after stent angioplasty, we generated a functional modified CD39 mRNA as a possible therapeutic agent for a novel stent coating and tested it using various *in vitro* assays.

### *In vitro* generation and purification of the CD39 mRNA

**Fig 1:**
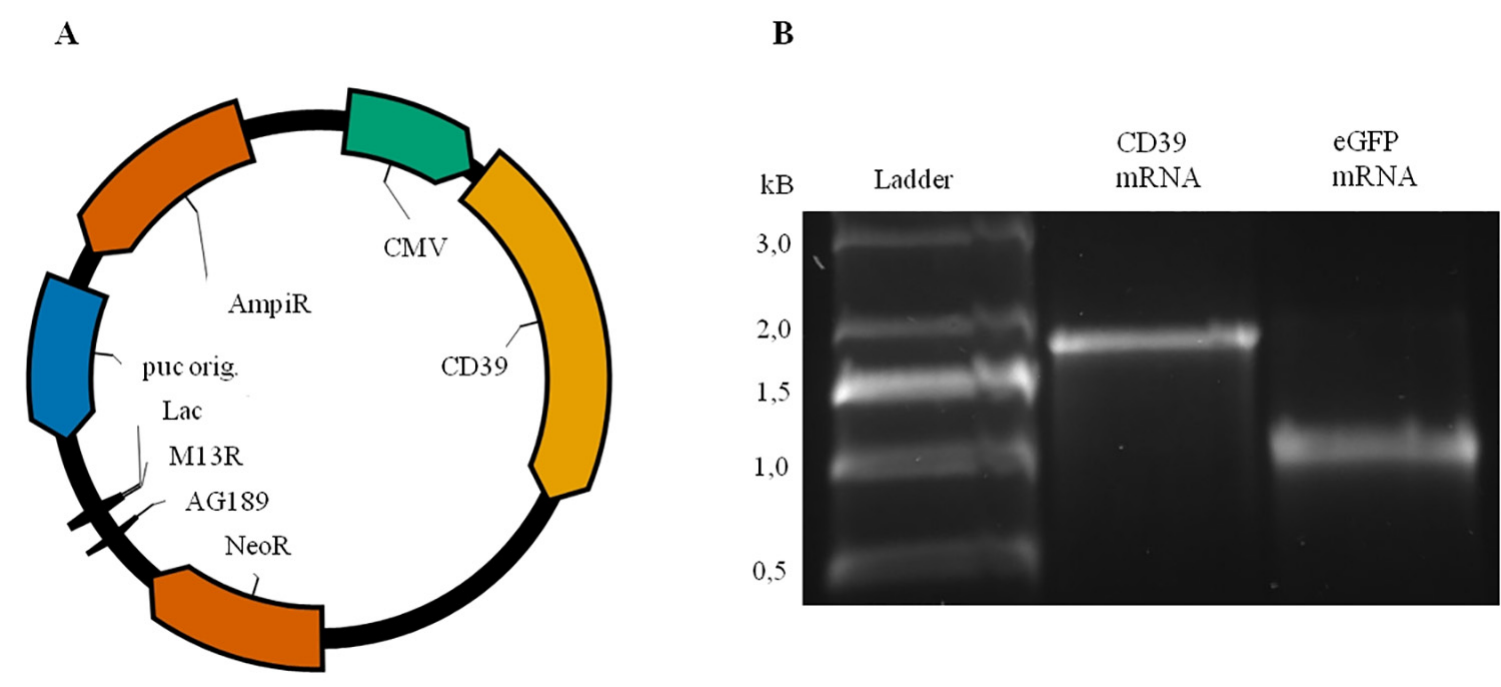
Vector map, generation and purification of CD39 mRNA. (A) Vector map of the pcDNA 3.3 plasmid containing the ENTDP-1/CD39 construct for mammalian expression. (B) Electrophoresis with a 1% agarose-TBE gel of the purified CD39 mRNA (1533 bp). eGFP mRNA (993 bp) was used as a loading control. 0.5–10 kB RNA ladder.

For mRNA generation, the gene of interest, i.e. CD39 or eGFP as a control, was cloned into the pcDNA 3.3 vector (Fig 1A) and the templates were amplified through PCR by using a specific primer pair [14]. Afterwards, the DNA was transcribed *in vitro* to mRNA and the mRNA purity was analyzed on an 1% agarose-TBE gel. As shown in Fig 1B, purified CD39 and eGFP mRNAs with the expected size of 1533 bp and 793 bp, respectively were successfully generated.

### *In vitro* transfection with CD39 mRNA induces overexpression of CD39 in HEK293 cells, PAE cells and ECs

**Fig 2:**
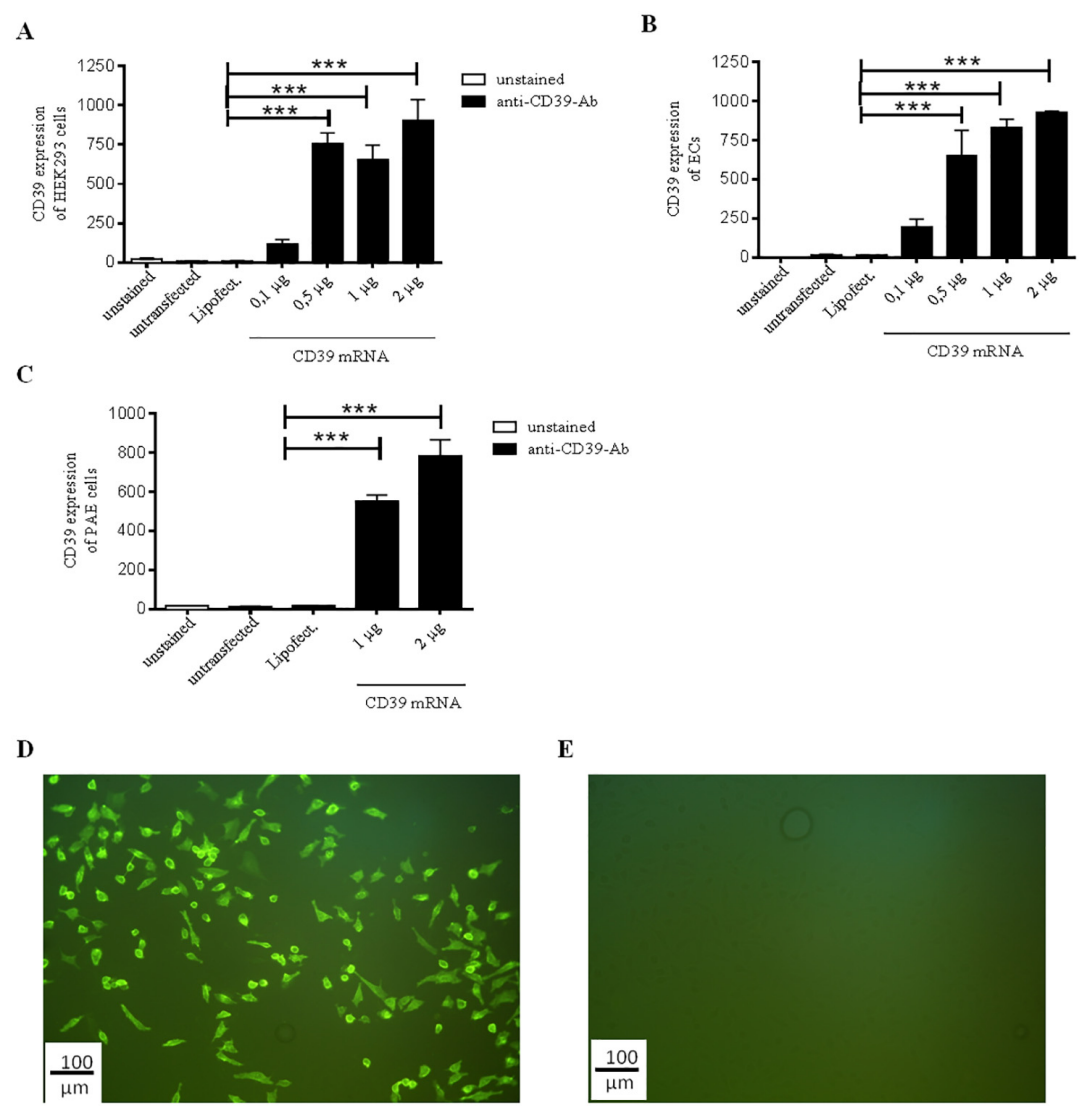
Expression of CD39 induced by *in vitro* transfection of HEK293 cells and ECs with the generated CD39 mRNA. Flow cytometric analyses of transfected HEK293 cells (A) human ECs (B) and immortalized porcine endothelial cells (PAE; C) with different CD39 mRNA concentrations. For control untreated and Lipofectamin-treated cells were used. The cells were labeled with a CD39-FITC antibody 24 h after transfection. Each experiment was performed at least 3 times. Data are given as means and SEM and were compared using one-way ANOVA (A & B) or RM-ANOVA (C) with Bonferroni´s multiple comparison test. ***p < 0.001. Representative fluorescence microscopic images of CD39 mRNA transfected (C, 2 μg CD39 mRNA) and untransfected (D) HEK293 cells.

In order to induce overexpression of functional CD39, HEK293 cells, PAE cells and ECs were transfected with different concentrations of the generated CD39 mRNA. After 24 h, 5 days and 7 days, the expression of CD39 of HEK293 cells was measured using flow cytometry. Compared to untransfected HEK293 cells and cells treated with Lipofectamin only, 0.5 μg of the CD39 mRNA was sufficient to induce a significant increase of the protein expression 24 h after transfection (***p< 0.001; Fig 2A). Using higher mRNA concentrations of 1 μg and 2 μg did not significantly increase the amount of CD39 protein expression on HEK293 cells when compared to the use of 0.5 μg CD39 mRNA. Similar results were found 24 h after *in vitro* transfection of primary human ECs (Fig 2B), while using 2 μg CD39 mRNA induced a slightly higher CD39 expression in these cells when compared to 0.5 μg (***p< 0.001). An amount of 0.5 μg of the CD39 mRNA was still sufficient to induce CD39 expression in the ECs.

With regard to future *in vivo* testing of the CD39 mRNA in a stent angioplasty model in pigs, immortalized porcine ECs (PAE) were also transfected with various concentrations of CD39 mRNA. Our data show that similar to human ECs, a significant increase in CD39 expression is achieved using 0.5 μg and 1 μg of mRNA/ 150.000 PAEs (Fig 2C) (***p< 0.001).

Additionally, the expression of CD39 in HEK293 cells was analyzed using the fluorescence microscopy. A distinct fluorescent signal was detected 24 h after transfection with 0.5 μg, 1 μg or 2 μg CD39 mRNA compared to non-transfected control cells (Fig 2D and 2E).

By flow cytometric analyzes we could also show that a single transfection with 0.5 μg or 1 μg CD39 mRNA is still sufficient to induce a significant increase in CD39 expression after 5 and 7 days compared to the controls (S1 Fig).

### CD39 mRNA transfection does not significantly decrease cell viability or increase pro-inflammatory cytokine release

**Fig 3:**
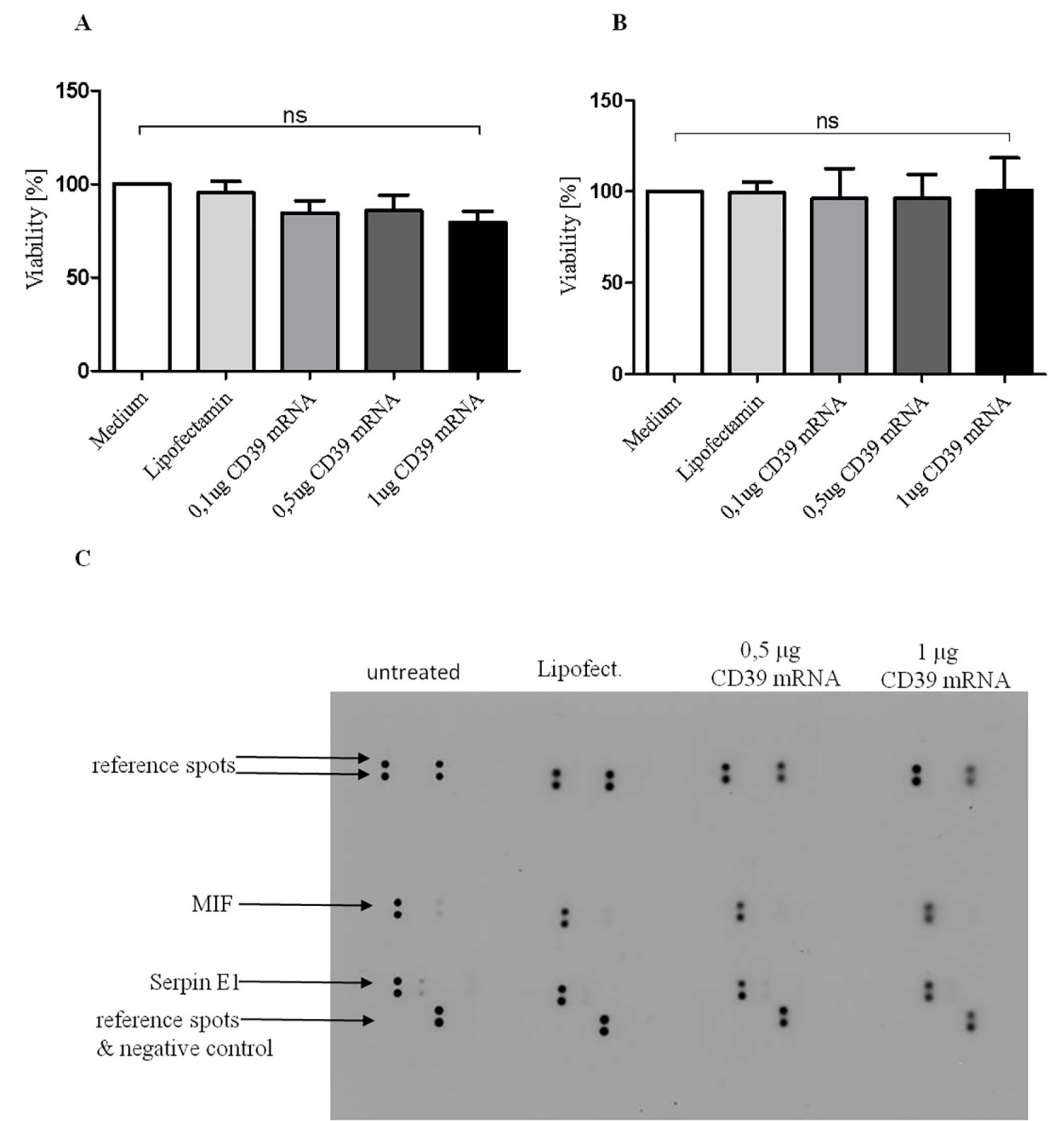
Overexpression of CD39 has no influence on the viability of the mRNA transfected HEK293 cells and the release of pro-inflammatory cytokines. An MTT assay was performed after 24 h (A) and 48 h (B) to analyze the cell viability in HEK293 cells after transfection with CD39 mRNA. (C) Detection of various cytokines released from untransfected and transfected HEK293 cells with 0.5 μg and 1 μg CD39 mRNA using a Proteome Profiler ^TM^ Array. The supernatant of untreated and Lipofectamin-treated HEK293 cells were used as control. Data are given as means and SEM and were compared using one-way ANOVA with Bonferroni´s multiple comparison test; ns: not significant.

We could also show via an MTT assay that CD39 mRNA transfection has no influence on the cell viability of HEK293 cells (Fig 3A and 3B). To get a first idea on whether the transfection of CD39 mRNA has an effect on cellular processes like inflammation, immunity, gene expression, cellular growth and migration, a human cytokine assay was performed (Proteome Profiler^TM^ Array) showing the relative expression levels of 36 different cytokines. Selected capture antibodies on a nitrocellulose membrane detect the suitable biotinylated antibody, which was mixed with the sample. Through a Streptavidin-HRP and chemiluminescent detection light is produced at each spot in proportion to the amount of cytokine bound.

The data indicate that 0.5 μg and 1 μg CD39 mRNA/ 150.000 HEK293 cells have no effect on the level of inflammatory cytokines compare to the controls. In all samples, including the control samples, however, MIF (macrophage migration inhibitory factor) and Serpin E1 (plasminogen activator inhibitor 1) could be detected in higher amounts (Fig 3C and S2 Fig).

### Evaluation of the functionality of the expressed CD39

**Fig 4:**
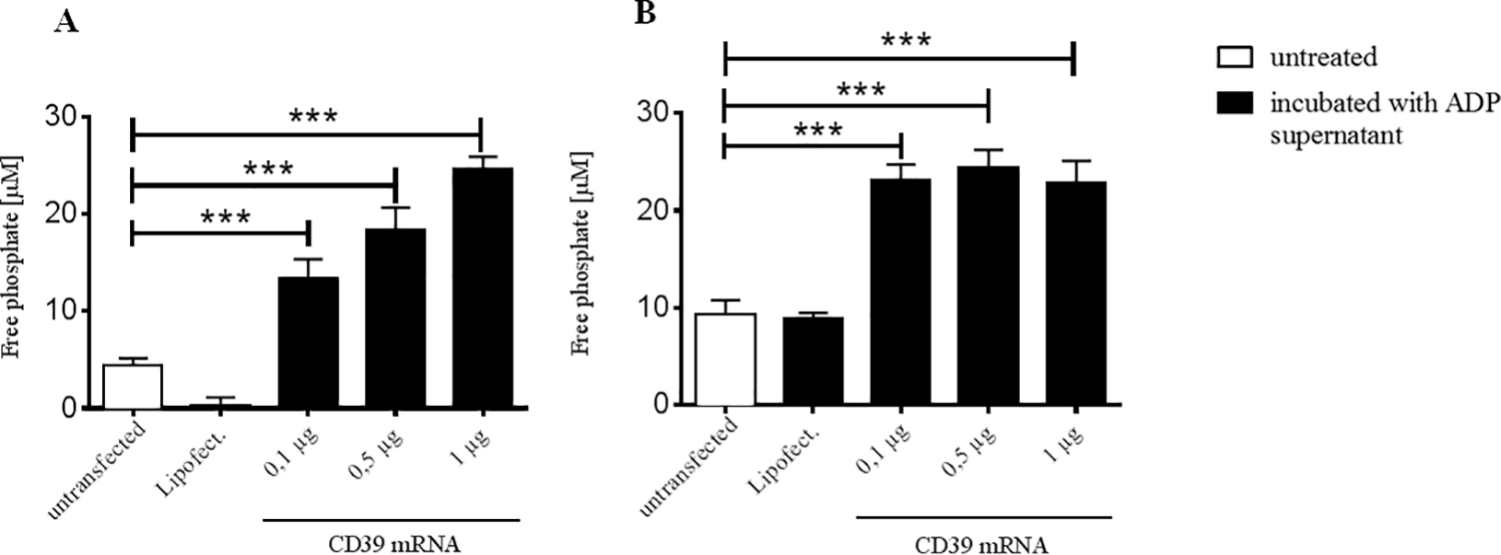
The expressed CD39 protein in HEK293 cells and ECs is highly functional in hydrolyzing ADP. CD39-transfected HEK293 cells (A) and human ECs (B) as well as untreated and Lipofectamin-treated cells were incubated with 200 μl ADP for 10 min at 37°C. Free phosphate was measured after incubating with 20 μl working solution in a plate reader at 620 nm absorbance. Data are given as means and SEM and were compared using RM-ANOVA with Bonferroni´s multiple comparison test. ***p < 0.001.

To investigate the functionality of the overexpressed CD39 protein, we measured phosphate liberated from hydrolyzed ADP. HEK293 cells and ECs were transfected with different concentrations of the generated CD39 mRNA and incubated with ADP after 24 h. Compared to untransfected cells and HEK293 cells treated with Lipofectamin, transfection with 0,1 μg of the CD39 mRNA was sufficient to induce a significant increase in free phosphate concentrations (***p< 0.001; Fig 4A). Higher mRNA concentrations of 0.5 μg and 1 μg showed also a slightly higher increase of free phosphate. Similar results were obtained after CD39 mRNA transfection of human ECs (***p< 0.001; Fig 4B).

**Fig 5:**
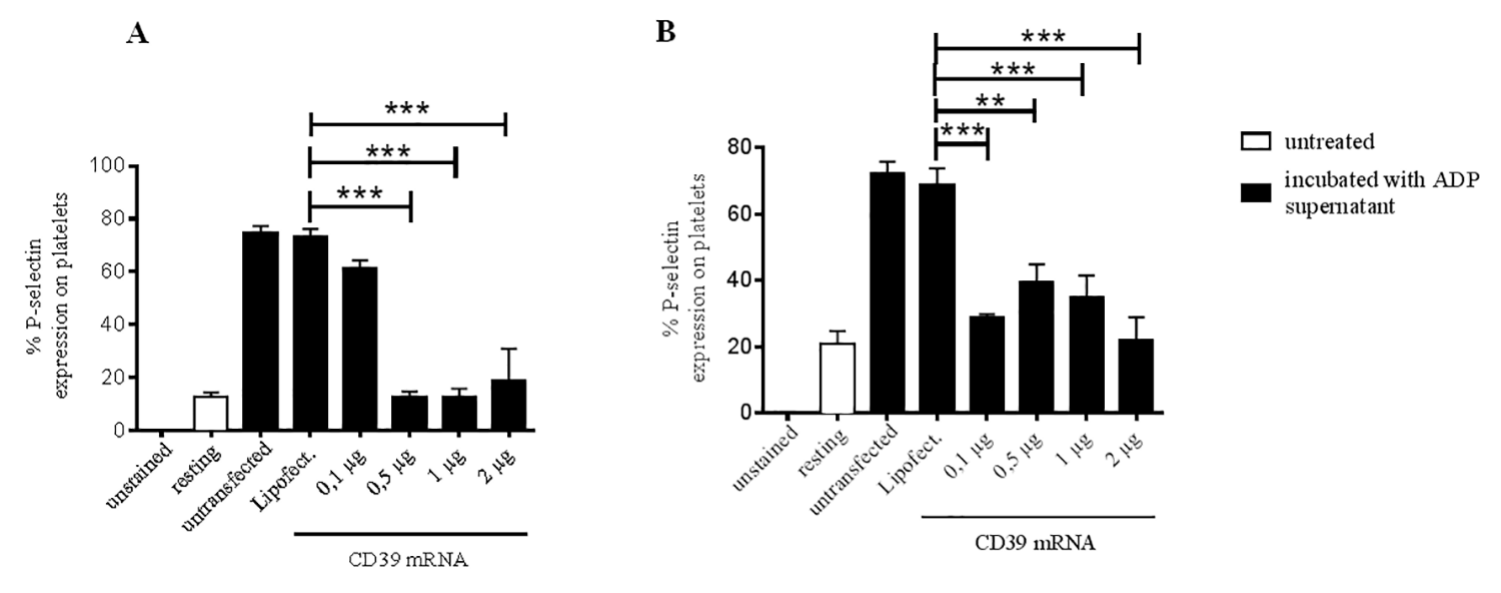
Overexpression of CD39 in HEK293 cells and endothelial cells significantly decreases platelet activation *in vitro*. CD39-transfected HEK293 cells (A) and EC cells (B) as well as untreated and Lipofectamin-treated cells were incubated with 20 μl ADP for 10 min at 37°C. Afterwards, the supernatants were incubated with PRP and platelet activation induced by non-hydrolyzed ADP was detected using an anti-CD62P-PE antibody in flow cytometry. Data are given as means and SEM and were compared using one-way ANOVA with Bonferroni´s multiple comparison test. **p < 0.01; ***p < 0.001.

Furthermore, the efficiency in inhibiting ADP-mediated platelet activation was measured with untransfected, Lipofectamin-treated and CD39 mRNA transfected cells. First PRP was treated with ADP supernatant, which was beforehand incubated with HEK293 cells and ECs. Afterwards, platelet activation was investigated by analyzing the expression of the cell adhesion molecule P-selectin on the platelet surface, which is a reliable indicator for platelet degranulation and thus platelet activation. Our data show that ADP supernatant incubated with CD39 mRNA-transfected HEK293 cells leads to a significant decrease in P-selectin expression on platelets at concentration of 0.5 μg CD39 mRNA (***p< 0.001; Fig 5A). More particularly, the ADP supernatant incubated with untransfected or Lipofectamin-treated HEK293 cells induced a P-selectin expression in 74.6 ±7.4 and 73.36 ± 7.5% of the platelets, respectively. HEK293 cells transfected with 0.5 μg mRNA efficiently hydrolyzed ADP, since a P-selectin expression in only 12.7 ± 3.2% of the platelets was measured. This is comparable to resting platelets (12.6 ± 4.6%) concluding that 0.5 μg mRNA is sufficient to effectively hydrolyze ADP.

Similar results were found after *in vitro* transfection of primary human ECs. Here CD39 expression induced by only 0.1 μg mRNA was sufficient to hydrolyze ADP so that a P-selectin expression in only 28.7 ± 1.9% of the platelets was found (Fig 5B).

This result is comparable to a P-selectin expression found on resting platelets (20.9 ± 9.4%). In contrast, ADP supernatant incubated with Lipofectamin-treated ECs induced a significantly higher P-selectin expression in platelets (***p< 0.001).

To recapitulate, we have shown that the expression of CD39 in HEK293 cells and ECs is highly efficient in hydrolyzing ADP and thus significantly inhibits ADP-mediated platelet activation.

### Indirect transfection of HEK293 cells via a PLGA-based CD39 mRNA coating

**Fig 6:**
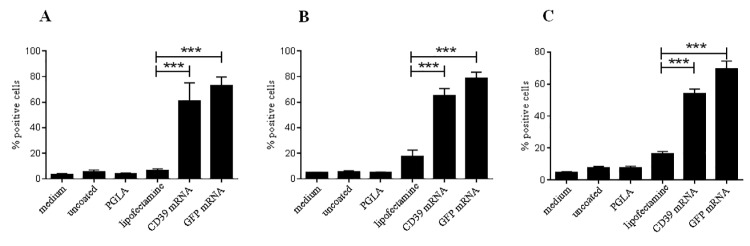
CD39 mRNA coating on PLGA slides significantly induces an expression in HEK293 cells. Thermanox slides were coated with PLGA and CD39 mRNA/ Lipofectamin complexes. A coating of eGFP/ Lipofectamin was used as control. After incubation of the slides with HEK293 cells the expression of CD39 and eGFP was measured by flow cytometry after 24 h (A), 48 h (B) and 72 h (C). Data are given as means and SEM compared using one-way ANOVA with Bonferroni´s multiple comparison test. ***p < 0.001.

In order to develop a bioactive stent coating, which allows local delivery of mRNA and transfection of ECs in vivo, the generated CD39 mRNA was first coated on thermanox plastic slides using the biodegradable compound PLGA. Flow cytometric analyzes of the HEK293 cells after incubation with the CD39 mRNA/PLGA covered thermanox slides showed that the CD39 mRNA is released form the PLGA coating, whereby a significant increase in CD39 expression was detectable after 24 h, 48 h and 72 h (***p< 0.001; Fig 6).

## Discussion

In this study a functional modified mRNA encoding the ecto-ADPase CD39 has been generated, which could be used as a novel agent for a bioactive stent coating to protect patients against stent angioplasty-associated complications.

Therapeutic strategies using a specific mRNA instead of DNA provide a novel attractive approach, which allows a direct expression of a desired protein within the target cells without affecting the genome itself [19,20].

We could demonstrate that the *in vitro* transfection of CD39 mRNA in HEK293 as well as in human and porcine endothelial cells is highly effective in inducing CD39 overexpression in the cells. In further experiments, the functionality of the generated CD39 mRNA was validated by measuring free phosphate after hydrolysis of ADP by transfected HEK293 cells and ECs. Our current findings also confirm previous experiments performed by Kaczmarek et al. [21], who also showed that a transient transfection of COS-7 cells with a pcDNA-CD39 plasmid results in the expression of active CD39, which also inhibits platelet aggregation in response to ADP [21]. In our study, we could also demonstrate by flow cytometric analyses that CD39 inhibits ADP-mediated platelet activation as indicated by a decrease of P-selectin on the surface of platelets. Our generated CD39 appears to have a strong anti-platelet effect and thus we assume that locally applied CD39 mRNA could reduce thrombotic complications after stent angioplasty.

There is increasing evidence that stent design, geometry, biocompatibility of the metal or surface coating may have an effect on the healing processes after stent implantation [22,23]. The introduction of drug eluting stents in 2002 showed a promising effect in decreasing stent thrombosis, neointimal proliferation and restenosis [6,8]. They are primary covered with immunosuppressive and antiproliferative drugs like sirolimus and paclitaxel [3,24]. However, very late in-stent-restenosis (IRS), delayed arterial healing, inflammation and endothelial dysfunction seem to be commonly linked to DES failure [6,25]. The treatment of patients with DES-associated ISR has appeared to be particularly challenging showing patients with drug resistance, cytotoxicity-, and local hypersensitivity reactions as well as a higher risk of infarction [8,24]. Furthermore, anti-platelet drugs have been a major cause to increase the risk of bleeding complications [26]. Therefore, today´s trends are towards generation of a novel biocompatible stent design and stent surface coating to minimalize medication and to promote the healing process after surgery. A possible stent design could be realized by Cui et al., who generated a stent coating based on antibodies to endoglin, which inhibit neointimal formation of porcine coronary arteries due to enhanced reendothelialization [27].

Previous studies investigating the effects of CD39 reveal its major effects as a natural pharmacologically agent because of its anti-thrombotic and anti-inflammatory potential [10,11,21,28]. In a murine model of ischemic stroke the administration of solCD39, a generated recombinant soluble form of CD39, showed that the frequency of stroke can be reduced without increasing intracerebral hemorrhage [28]. Additionally, CD39 shows to have similar antiplatelet effects to that observed with abciximab, which is also used in patients with ISR, but with low clinical benefit [10].

**Fig 7:**
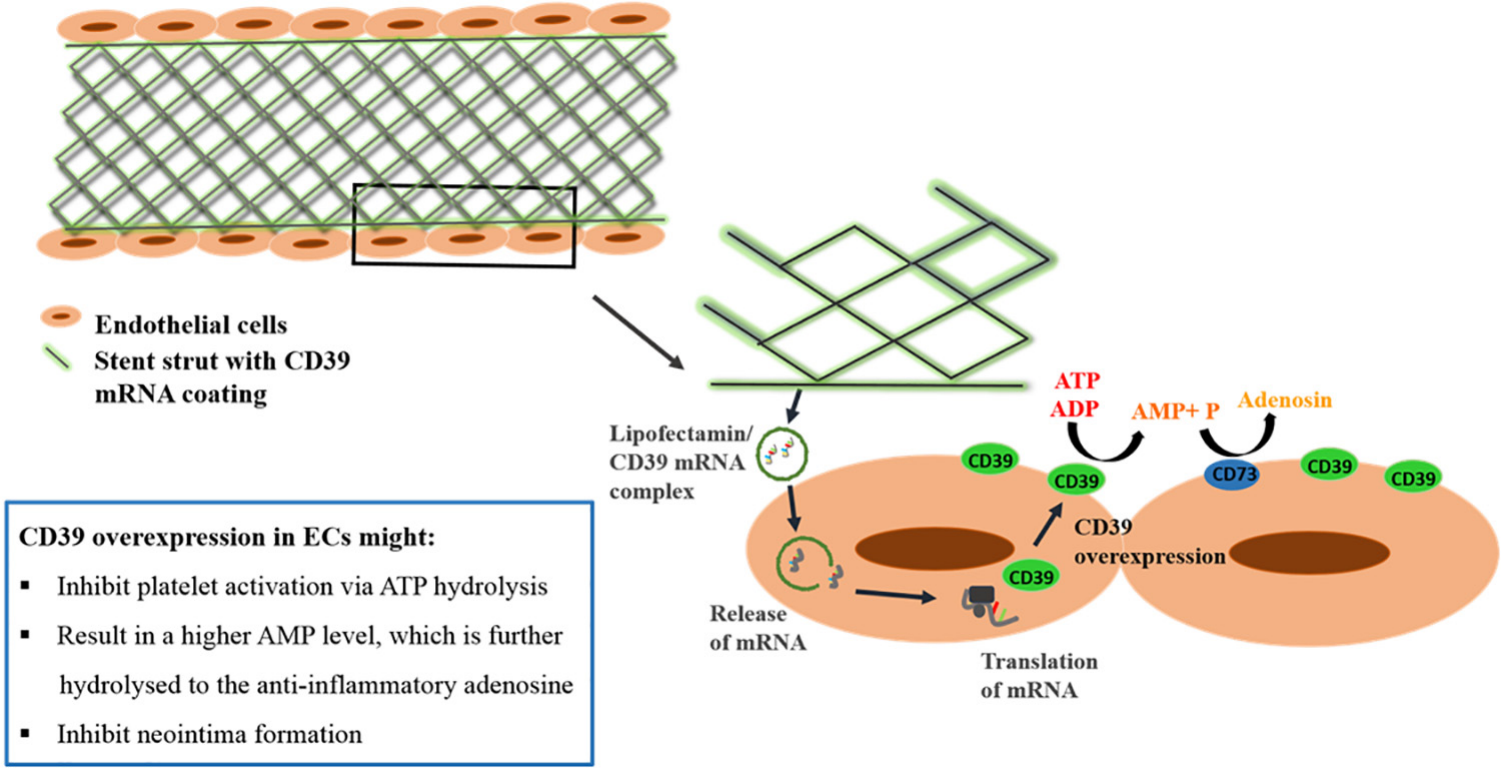
The hypothetical effect of a CD39 mRNA-stent coating on endothelial cells. Implantation of a CD39 mRNA-coated stent into an atherosclerotic blood vessel will result in transfection of endothelial cells, which in turn express the CD39 protein. The overexpression of the CD39 protein will result in augmented hydrolysis of ATP and ADP and hence may inhibit platelet activation and neointima formation as well as support anti-inflammatory processes.

Furthermore, the activity of vascular CD39 to hydrolyze extracellular nucleotides is lost due to the denudation of the endothelium following (stent) angioplasty. The increased local concentration of the nucleotides ATP and ADP leads to oxidative stress, release of pro-inflammatory mediators, the activation of platelets and hence thrombotic complications. [9]. Moreover, extracellular nucleotides are mainly involved in the process of neointima hyperplasia, the proliferation and migration of vascular smooth muscle cells, which is the main cause of restenosis. Koziak et al. [9] as well as Drosopoulos et al. [12] could show that increased CD39 activity prevents nucleotid-induced proliferation in VSMC (vascular smooth muscle cells) and intimal hyperplasia and therefore is involved in neointima formation. Consequently, we hypothesize that a CD39 mRNA coating at the outside surface of a stent could directly induce the local expression of CD39 to replace its lost function on the injured vessel wall (Fig 7). We hypothesize that the surrounding endothelial cells and SMCs will take up the CD39 mRNA/Lipofectamin complexes, translate the mRNA and express active CD39 protein on the cell surface. Thus, CD39 may support healing processes and the integrity of the endothelium by hydrolyzing pro-thrombotic and pro-inflammatory nucleotides thereby reducing thrombotic events. Additionally, CD39 expression will also regulate SMC proliferation, a common contributor to restenosis.

## Conclusion

In conclusion, our data show that endothelial cells successfully express active CD39 after direct or indirect *in vitro* transfection using modified CD39 encoding mRNA within the context of a new mRNA therapy approach.

Next to inhibition of platelet activation, the local release of CD39 mRNA in atherosclerotic blood vessels would support the integrity of the endothelium and inhibit proliferation of smooth muscle cells caused by extracellular nucleotides.

Overall, the study presents a completely innovative and promising therapeutic concept to most likely reduce the risk of stent angioplasty-associated complications thereby increasing the safety of patients.

While we envision a titanium stent spray coated with the CD39 mRNA, optimization of the CD39 mRNA coating as well as *in vivo* experiments with stents are needed to confirm the functionality, effectiveness and release kinetics of the CD39 mRNA as potential stent coating.

## Supporting Information

S1 Fig The expression of CD39 in HEK239 cells is still significant after 5 and 7 days after *in vitro* transfection. Flow cytometric analyses of transfected HEK293 cells with different CD39 mRNA concentrations. For control untreated and Lipofectamin-treated cells were used. The cells were labeled with CD39-PE antibody (BD Bioscience) and analyzed 5 days (A) and 7 days (B) after the transfection.Data are given as means and SEM compared using one-way ANOVA with Bonferroni´s multiple comparison test. ***p < 0,001.
(TIF)

S2 Fig Overexpression of CD39 has no influence on the release of pro-inflammatory cytokines. Detection of various cytokines released from untransfected and transfected HEK293 cells with 0.5 μg and 1 μg CD39 mRNA. The supernatant of untreated and Lipofectamin-treated HEK293 cells were used as control. Data analysis was performed by measuring the pixel density in each spot of the array (compare Fig 3C) and calculating the data with the image processing program ImageJ.
(TIF)

## References

[1] Hansson GK, Libby P (2006) The immune response in atherosclerosis: a double-edged sword. Nat Rev Immunol 6: 508–519. 16778830 10.1038/nri1882

[2] Weber C, Noels H (2011) Atherosclerosis: current pathogenesis and therapeutic options. Nat Med 17: 1410–1422. 10.1038/nm.2538 22064431

[3] Fortier A, Gullapalli V, Mirshams RA (2014) Review of biomechanical studies of arteries and their effect on stent performance. IJC Heart & Vessels.

[4] Simard T, Hibbert B, Ramirez FD, Froeschl M, Chen YX, O'Brien ER (2014) The evolution of coronary stents: a brief review. Can J Cardiol 30: 35–45. 10.1016/j.cjca.2013.09.012 24286961

[5] Erlinge D (1998) Extracellular ATP: a growth factor for vascular smooth muscle cells. Gen Pharmacol 31: 1–8. 9595270 10.1016/s0306-3623(97)00420-5

[6] Rodriguez-Granillo A, Rubilar B, Rodriguez-Granillo G, Rodriguez AE (2011) Advantages and disadvantages of biodegradable platforms in drug eluting stents. World J Cardiol 3: 84–92. 10.4330/wjc.v3.i3.84 21499496 PMC3077815

[7] Burnstock G (2002) Purinergic signaling and vascular cell proliferation and death. Arterioscler Thromb Vasc Biol 22: 364–373. 11884276 10.1161/hq0302.105360

[8] Alfonso F, Byrne RA, Rivero F, Kastrati A (2014) Current treatment of in-stent restenosis. J Am Coll Cardiol 63: 2659–2673. 10.1016/j.jacc.2014.02.545 24632282

[9] Koziak K, Bojakowska M, Robson SC, Bojakowski K, Soin J, Csizmadia E, et al (2008) Overexpression of CD39/nucleoside triphosphate diphosphohydrolase-1 decreases smooth muscle cell proliferation and prevents neointima formation after angioplasty. J Thromb Haemost 6: 1191–1197. 10.1111/j.1538-7836.2008.03019.x 18485080 PMC2761653

[10] Sampram ES, Ouriel K (2004) In vitro verification of antithrombotic effect of recombinant soluble nucleotide triphosphate diphosphohydrolase 1. J Vasc Interv Radiol 15: 379–384. 15064342 10.1097/01.rvi.0000121409.46920.b8

[11] Marcus AJ, Broekman MJ, Drosopoulos JH, Islam N, Alyonycheva TN, Safier LB, et al (1997) The endothelial cell ecto-ADPase responsible for inhibition of platelet function is CD39. J Clin Invest 99: 1351–1360. 9077545 10.1172/JCI119294 PMC507951

[12] Drosopoulos JH, Kraemer R, Shen H, Upmacis RK, Marcus AJ, Musi E (2010) Human solCD39 inhibits injury-induced development of neointimal hyperplasia. Thromb Haemost 103: 426–434. 10.1160/TH09-05-0305 20024507 PMC2847853

[13] Dwyer KM, Robson SC, Nandurkar HH, Campbell DJ, Gock H, Murray-Segal LJ, et al (2004) Thromboregulatory manifestations in human CD39 transgenic mice and the implications for thrombotic disease and transplantation. J Clin Invest 113: 1440–1446. 15146241 10.1172/JCI19560 PMC406523

[14] Avci-Adali M, Behring A, Keller T, Krajewski S, Schlensak C, Wendel HP (2014) Optimized conditions for successful transfection of human endothelial cells with in vitro synthesized and modified mRNA for induction of protein expression. J Biol Eng 8: 8 10.1186/1754-1611-8-8 24581116 PMC3975882

[15] Kormann MSD, Hasenpusch G, Aneja MK, Nica G, Flemmer AW, Herber-Jonat S, et al (2011) Expression of therapeutic proteins after delivery of chemically modified mRNA in mice. Nat Biotech 29: 154–157. 10.1038/nbt.1733 21217696

[16] Tavernier G, Andries O, Demeester J, Sanders NN, De Smedt SC, Rejman J (2011) mRNA as gene therapeutic: how to control protein expression. J Control Release 150: 238–247. 10.1016/j.jconrel.2010.10.020 20970469

[17] Avci-Adali M, Kobba J, Neumann B, Lescan M, Perle N, Wilhelm N, et al (2013) Application of a rotating bioreactor consisting of low-cost and ready-to-use medical disposables for in vitro evaluation of the endothelialization efficiency of small-caliber vascular prostheses. J Biomed Mater Res B Appl Biomater 101: 1061–1068. 10.1002/jbm.b.32916 23653116

[18] Krajewski S, Rheinlaender J, Ries P, Canjuga D, Mack C, Scheideler L, et al (2014) Bacterial interactions with proteins and cells relevant to the development of life-threatening endocarditis studied by use of a quartz-crystal microbalance. Anal Bioanal Chem 406: 3395–3406. 10.1007/s00216-014-7769-9 24705960

[19] Zohra FT, Chowdhury EH, Akaike T (2009) High performance mRNA transfection through carbonate apatite–cationic liposome conjugates. Biomaterials 30: 4006–4013. 10.1016/j.biomaterials.2009.02.050 19410288

[20] Zohra FT, Chowdhury EH, Tada S, Hoshiba T, Akaike T (2007) Effective delivery with enhanced translational activity synergistically accelerates mRNA-based transfection. Biochemical and Biophysical Research Communications 358: 373–378. 17475211 10.1016/j.bbrc.2007.04.059

[21] Kaczmarek E, Koziak K, Sevigny J, Siegel JB, Anrather J, Beaudoin AR, et al (1996) Identification and characterization of CD39/vascular ATP diphosphohydrolase. J Biol Chem 271: 33116–33122. 8955160 10.1074/jbc.271.51.33116

[22] McLean DR, Eiger NL (2002) Stent design: implications for restenosis. Rev Cardiovasc Med 3 Suppl 5: S16–22. 12478231

[23] Nilsson PH, Ekdahl KN, Magnusson PU, Qu H, Iwata H, Ricklin D, et al (2013) Autoregulation of thromboinflammation on biomaterial surfaces by a multicomponent therapeutic coating. Biomaterials 34: 985–994. 10.1016/j.biomaterials.2012.10.040 23137394 PMC4705352

[24] Rha SW (2014) Duration of dual antiplatelet treatment in the era of next generation drug-eluting stents. World J Cardiol 6: 148–153. 10.4330/wjc.v6.i4.148 24772255 PMC3999335

[25] Khan W, Farah S, Domb AJ (2012) Drug eluting stents: developments and current status. J Control Release 161: 703–712. 10.1016/j.jconrel.2012.02.010 22366546

[26] Patrono C, Andreotti F, Arnesen H, Badimon L, Baigent C, Collet JP, et al (2011) Antiplatelet agents for the treatment and prevention of atherothrombosis. Eur Heart J 32: 2922–2932. 10.1093/eurheartj/ehr373 22019823

[27] Cui S, Liu JH, Song XT, Ma GL, Du BJ, Lv SZ, et al (2014) A novel stent coated with antibodies to endoglin inhibits neointimal formation of porcine coronary arteries. Biomed Res Int 2014: 428619 10.1155/2014/428619 24883312 PMC4026940

[28] Marcus AJ, Broekman MJ, Drosopoulos JH, Islam N, Pinsky DJ, Sesti C, et al (2003) Metabolic control of excessive extracellular nucleotide accumulation by CD39/ecto-nucleotidase-1: implications for ischemic vascular diseases. J Pharmacol Exp Ther 305: 9–16. 12649347 10.1124/jpet.102.043729

