# Supplementary figures and images for: In vitro Study of a Novel Stent Coating Using Modified CD39 Messenger RNA to Potentially Reduce Stent Angioplasty-Associated Complications
Source: PLoS One. 2015 Sep 18;10(9):e0138375. doi: 10.1371/journal.pone.0138375 (PMC4575070; doi:10.1371/journal.pone.0138375)

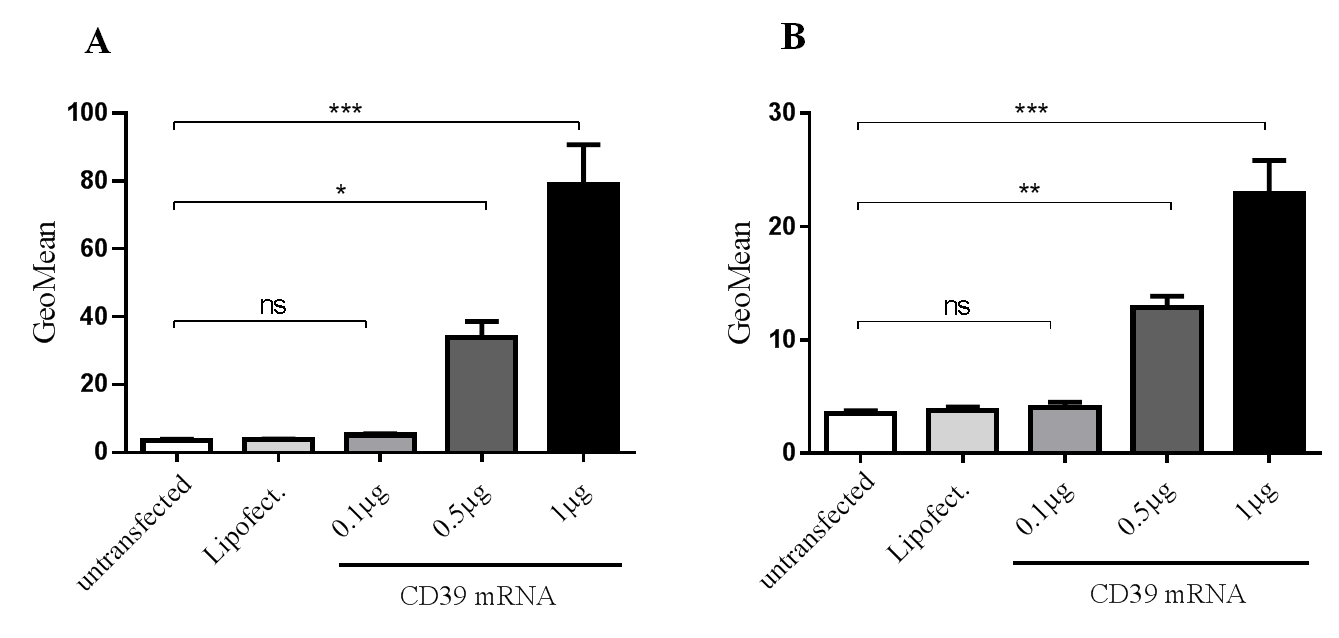

Supplement: S1 Fig — Flow cytometric analyses of transfected HEK293 cells with different CD39 mRNA concentrations. For control untreated and Lipofectamin-treated cells were used. The cells were labeled with CD39-PE antibody (BD Bioscience) and analyzed 5 days (A) and 7 days (B) after the transfection.Data are given as means and SEM compared using one-way ANOVA with Bonferroni´s multiple comparison test. ***p < 0,001. (TIF) [file pone.0138375.s001.tif]

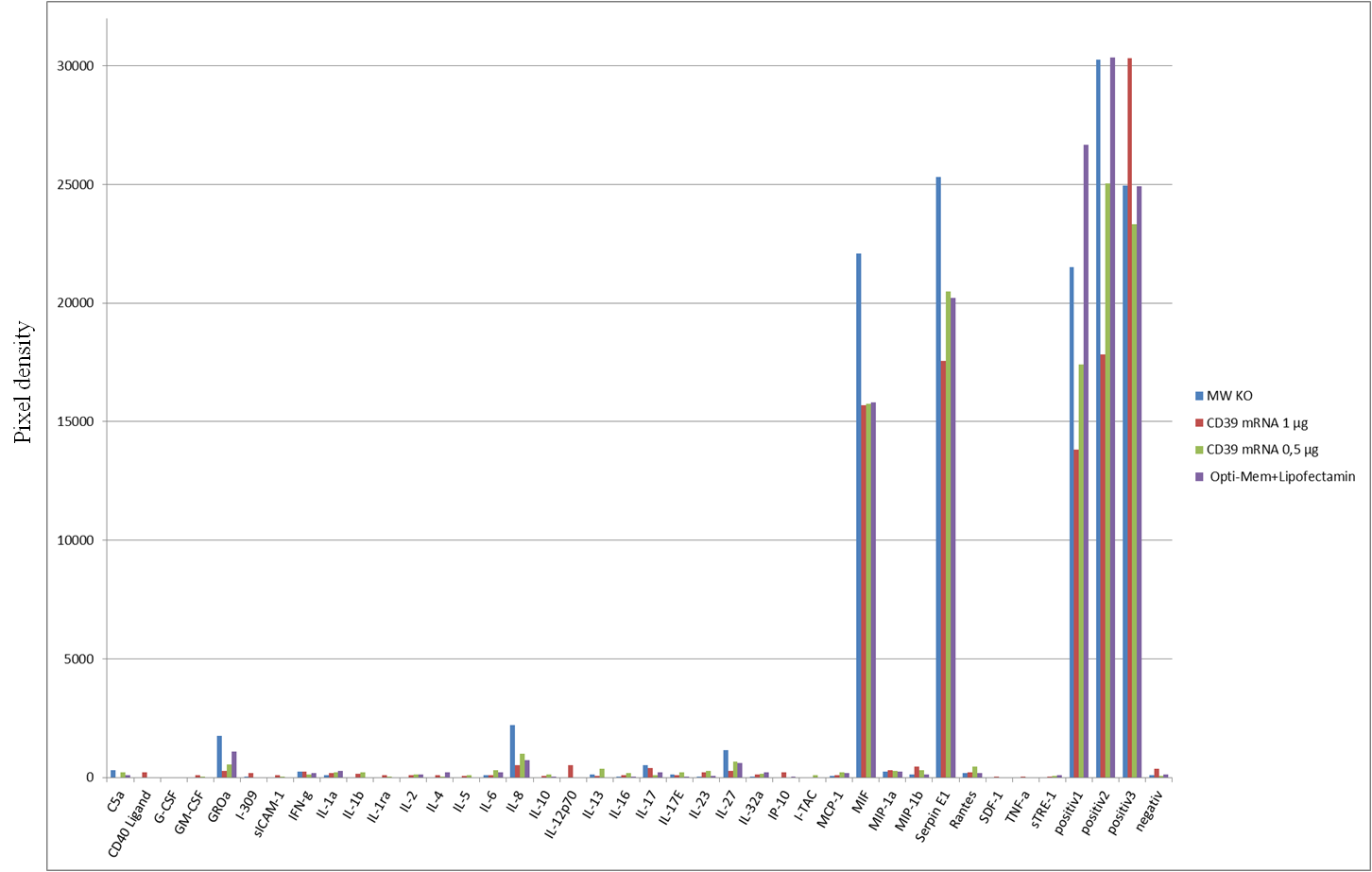

Supplement: S2 Fig — Detection of various cytokines released from untransfected and transfected HEK293 cells with 0.5 μg and 1 μg CD39 mRNA. The supernatant of untreated and Lipofectamin-treated HEK293 cells were used as control. Data analysis was performed by measuring the pixel density in each spot of the array (compare Fig 3C) and calculating the data with the image processing program ImageJ. (TIF) [file pone.0138375.s002.tif]
